# Plasma membrane damage by environmental materials enhances cell-cell fusion and impairs defense functions of macrophages

**DOI:** 10.3389/fimmu.2026.1898611

**Published:** 2026-09-16

**Authors:** Dandan Wu, Xing Zhang, Ruoning Wang, Amanda Livingston, Xinyi Lu, Eliane El Hayek, Xiang Xue, Aaron Neumann, Matthew Campen, Meilian Liu, Xuexian O. Yang

**Affiliations:** 1 Department of Molecular Genetics and Microbiology, University of New Mexico School of Medicine, Albuquerque, NM, United States; 2 National and Local Joint Engineering Laboratory of Animal Peptide Drug Development, College of Life Science, Hunan Normal University, Changsha, Hunan, China; 3 Department of Biochemistry and Molecular Biology, University of New Mexico School of Medicine, Albuquerque, NM, United States; 4 Department of Pharmaceutical Sciences, University of New Mexico College of Pharmacy, Albuquerque, NM, United States; 5 Department of Pathology, University of New Mexico School of Medicine, Albuquerque, NM, United States

**Keywords:** environmental material, host defense, macrophage, membrane damage, phagocytosis

## Abstract

**Introduction:**

Many environmental materials can activate macrophages and trigger inflammatory responses. However, whether and how these exposures alter macrophage function in host defense remains elusive.

**Methods:**

Bone marrow-derived macrophages were treated with various environmental materials, including laundry detergent, and their membrane damage was measured by Annexin V staining. Alterations in macrophage function were assessed by cell-cell fusion, in vitro phagocytosis, and in vivo clearance of Candida albicans.

**Results and Discussion:**

Many environmental hazards damage the plasma membranes of macrophages. This damage decreases macrophage phagocytosis but elevates cell-cell fusion. In vivo, airway exposure to laundry detergent impairs the recruitment of macrophages and other myeloid cells to the lung and dramatically dampens protective TH1 and TH17 cell responses, thereby increasing susceptibility to Candida infection in mice. Overall, our data indicate that exposure to environmental materials compromises macrophage membrane integrity and impairs host defense. These findings may aid in developing effective preventive and therapeutic strategies.

## Introduction

Both man-made and natural materials have been widely used since the Industrial Revolution. Many of these materials are harmful and pose risks to human health. Among environmental hazards, particulate matter (PM) is a particular concern due to its surface properties, including size, shape, and chemical composition. Air pollutants contain PM of various sizes, with PM2.5 and PM0.1 being especially harmful, as they can be inhaled deeply into the lower respiratory tract, traverse the epithelial barrier, and reach multiple organs. PM exposure significantly impacts human health, contributing to the development of respiratory, cardiovascular, and neurological diseases, as well as increasing the risk of certain cancers (1).

Tungsten (in its carbide form, or WC) and cobalt are hard metals widely used in cutting and drilling tools, abrasives, and wear-resistant coatings. Occupational exposure to hard metal dusts, such as WC and cobalt, has been associated with respiratory disorders, skin allergies, and even lung cancer in industrial workers (2–5). Carbon nanotubes—cylindrical nanostructures composed of carbon atoms—are valued for their exceptional mechanical, electrical, and thermal properties, making them attractive for applications in electronics, materials science, and medicine (6). However, certain types of carbon nanotubes, when inhaled, can induce inflammation, oxidative stress, and lung damage (7, 8). In addition to PM, soluble substances, such as surfactants and phosphates (components of common detergents), can be toxic upon ingestion or skin absorption (9–12). Inhalation of detergent residues can cause bronchoconstriction and airway hyperresponsiveness, increasing the risk of asthma attacks (13, 14). Chronic exposure to these chemicals may lead to adverse long-term health effects, including organ damage.

Macrophages play a crucial role in immune defense and tissue homeostasis, especially within the innate immune system. They are responsible for detecting, engulfing, and degrading pathogens, foreign particles, and cellular debris (15). Environmental materials, such as tungsten carbide, carbon nanotubes, and certain detergent components, have been shown to alter macrophage function, including phagocytosis, inflammatory response, and cytotoxicity (16, 17), potentially compromising host defense mechanisms. Macrophages not only act as individual cells but also form multinucleated giant cells (MGCs), which are functionally distinct from conventional macrophages (18–20). Based on their anatomical location, morphology, and function, MGCs can be classified into osteoclasts (specialized in bone remodeling), Langhans giant cells (LGCs; involved in infectious and non-infectious granulomatous diseases), and foreign body giant cells (FBGCs; present at the site of prosthetic implantation or medical device placement). Notably, occupational or environmental exposure to hard metals can cause hard metal lung disease, also known as giant cell interstitial pneumonia (21–25). Certain environmental materials can even act as pathogen carriers and increase susceptibility to infectious disease (26). While inflammation elicited by environmental materials is extensively studied, whether and how these materials contribute to macrophage dysfunction and shift disease susceptibility remain poorly understood.

A better understanding of the interactions between macrophages and environmental materials is critical for evaluating health risks and developing effective interventions. In this study, we demonstrate that various environmental substances—including laundry detergent, saponin (a natural surfactant known to influence cell membrane (27)), carbon nanotubes, WC, and kaolin—can cause plasma membrane damage in macrophages. This damage promotes MGC formation through a CD44-dependent mechanism. Moreover, detergent and saponin suppresses the production of reactive oxygen species (ROS) and other inflammatory mediators and impairs macrophage phagocytic activity. *In vivo*, airway exposure to detergent impairs antifungal immune responses in mice. Together, our findings suggest that environmental materials compromise the membrane integrity of macrophages and impair their protective immune function. These insights may help us develop novel preventative and therapeutic options.

## Materials and methods

### Mice

All mice were housed in the specific pathogen-free animal facility, and all procedures were conducted at the University of New Mexico Health Sciences Center in accordance with protocols approved by the Institutional Animal Care and Use Committee.

### Administration of laundry detergent and *Candida albicans* airway infection

Sex-matched C57BL/6 mice (6–8 weeks old) were intratracheally (i.t.) administered 40 μL of a commercially available liquid laundry detergent (1%; as described for induction of asthma (14)) or vehicle control (phosphate-buffered saline, PBS). Two hours later, the mice were i.t. challenged with 1 × 10^6^ C*. albicans in* 40 μL of PBS, daily for four consecutive days. The i.t. administration was performed under anesthesia with 2-3% isoflurane in oxygen for about 3 min. Mice were sacrificed one day after the final dose of *C. albicans*, and lung tissues and bronchoalveolar lavage fluid (BALF) were collected for further analysis. The group sizes were determined by power analysis. To reach statistical significance (*p* ≤ 0.05) in an unpaired T-test with 80% power, we need 5–6 animals per group to detect an effect size of 1.8. No significant differences were observed between male and female mice.

### Colony formation assay

One percent (10 μl out of 1 mL) of each BALF sample was plated onto a yeast extract peptone dextrose (YPD) agar plate and incubated at 30 °C overnight. Colonies were counted by size and morphology, with large white colonies identified as *C. albicans*.

### Generation and culture of BMDMs

C57BL/6 mice (6–8 weeks old) were euthanized by cervical dislocation. Femurs and tibias were harvested, and bone marrow (BM) cells were flushed from the medullary cavities. BM cells were cultured in complete RPMI-1640 medium (cRPMI, Corning) containing 2 mM L-glutamine (Gibco), 10% fetal bovine serum (FBS; Atlanta Biologicals), and 100 U/mL penicillin-streptomycin (Gibco) in the presence of 20 ng/mL recombinant mouse macrophage colony-stimulating factor (M-CSF; Biolegend). Cultures were incubated at 37˚C with 5% CO_2_ for 6–7 days to induce macrophage differentiation. BMDMs were harvested using Accutase^®^ Cell Detachment Solution (Biolegend). To form MGCs, BMDMs were supplied with 10 ng/mL IL-4 (PeproTech) in the presence of M-CSF (20 ng/mL) and cultured as above for 4 days (28).

### THP-1 differentiation

Human leukemia monocytic THP-1 cells were maintained in cRPMI medium at 37 °C with 5% CO_2_. 20,000 THP-1 cells were plated in a 96-well plate and differentiated into macrophages with 80 ng/mL phorbol 12-myristate 13-acetate (P1585, Sigma-Aldrich) for 48 h as described (29).

### Treatments with environmental materials and assessment of plasma membrane damage

BMDMs were treated with 12.5 μg/mL saponin (S7900, Sigma-Aldrich) or the vehicle for 10 min, 1:20,000 dilution of liquid laundry detergent (a common commercial brand; similar to that described (13)) or the vehicle for 2 h, or 10 μg/mL carbon nanotube (10–30 nm, #900-1201, SES Research), 10 μg/mL kaolin (K7375, Sigma-Aldrich), 10 μg/mL WC (150–200 nm, 778346, Sigma-Aldrich), or the vehicle overnight. The doses and times were chosen to result in significant membrane damage but limited cell death, based on the dose-response and time-course experiments shown in **Supplementary Figures 1**–**3**. The resulting cells were labeled with Alexa Fluor 488-conjugated Annexin V to identify plasma membrane disruption and propidium iodide (PI) to determine dead cells. Images were acquired using the EVOS FL Cell Imaging System (Thermo Fisher Scientific).

### Hematoxylin and eosin staining

BMDMs treated with saponin (12.5 μg/mL), carbon nanotubes (1.25 μg/mL), laundry detergent (1%), or PBS (vehicle) were cultured in the presence of M-CSF with or without IL-4 for 6 days. The resulting cells were fixed with 4% paraformaldehyde at room temperature for 10 min and stained with H&E according to standard protocols. Images were acquired using the EVOS FL Cell Imaging System (Thermo Fisher Scientific).

### Flow cytometry

Antibodies against CD45.2 (104), CD4 (RM4.5), CD11b (M1/70), CD11c (N418), Ly-6G (1AB-Ly6g), SiglecF (E50-2440), Gr-1 (RB6-8C5), IFNγ (XMG1.2), IL-17A (eBio17B7), CD86 (GL-1), MHC-II (M5/114.15.2) were purchased from eBioscience. Cells stained with the indicated markers were analyzed with an Attune NxT Flow Cytometer (Thermo Fisher Scientific), and the data were processed by Flowjo software (FlowJo, LLC).

### Culture and CFSE-labelling of *C. Albicans* and *Escherichia coli*

*C. albicans* SC5314 (ATCC, MYA-2876), recovered from a −80 °C glycerol stock, was grown in YPD medium (L22052801, US Biological Life Sciences) for 16 h at 30 °C in an orbital shaker at 250 rpm to mid-log phase. *E. coli* was cultured in LB medium for 16 h at 37 °C with orbital shaking at 250 rpm to mid-log phase. After being washed, *C. albicans* or *E. coli* were suspended and incubated in 10 µM CFSE (C34554, Invitrogen)-PBS in the dark at room temperature for 10 min. The cells were then washed with cold PBS, suspended in RPMI medium, and stored on ice for later use.

### Phagocytosis assay

BMDMs, pre-treated with detergent, saponin, or vehicle as above, were incubated with CFSE-labelled live *C. albicans* or *E. coli* [Macrophage to *C. albicans* (or *E. coli*) = 1:10] at 37˚C in an atmosphere containing 5% CO_2_. After 30-min co-culture, the plate was washed with cold PBS. The resulting BMDMs were fixed with 4% paraformaldehyde for 15 min and stained with DAPI and CD11b (M1/70) for 10 min. High-content microscopy with automated image acquisition and quantification was carried out using a Cellomics HCS scanner equipped with iDEV software (Thermo Fisher Scientific). All data collection, object assignments, identification of regions of interest, and analyses were performed by the software in an unsupervised manner.

### 2',7'-dichlorodihydrofluorescein diacetate (DCFH-DA) staining

2 × 10^5^ BMDMs were plated in a 24-well plate and cultured overnight. The next day, the cells were treated with 12.5 μg/mL saponin, 1% liquid laundry detergent, or the vehicle for 10 min. After being washed with cold PBS, the resulting cells were stained with DCFH-DA (Cayman Chemical) and Hoechst or DAPI at 37 °C for 30 min. Following washes, the cells were kept in PBS, and images were acquired using a Cellomics HCS scanner equipped with iDEV software (Thermo Fisher Scientific). The cells were then lysed in 200 μL of radioimmunoprecipitation assay (RIPA) buffer, and 100 μL of each lysate was transferred into a black 96-well plate for measuring fluorescence intensity using a fluorescence microplate reader at an excitation wavelength of 485 nm and an emission wavelength of 530 nm. Protein concentrations were detected in 1 μL of lysates diluted in 100 μL of 1× protein assay solution using the Quick Start™ Bradford 1× Dye Reagent (Bio-Rad). Fluorescence intensities were normalized to the protein concentrations as described (30).

### Generation of CRISPR knockout

CRISPR KO of CD44 in macrophages was performed as described (31). Briefly, the gRNA (Mm.Cas9.CD44.1.AA,

/AltR1/rUrA rUrGrG rUrArA rCrCrG rGrUrC rCrArU rCrGrA rGrUrU rUrUrA rGrArG rCrUrA rUrGrC rU/AltR2/, Integrated DNA Technologies) were mixed with Cas9 (IDT Alt-R S.p. Cas9 Nuclease V3) at a molar ratio of 2:1 and incubated at room temperature for 20 min. 5 × 10^6^ *in-vitro* differentiated BMDMs were washed and resuspended in 20 μL of P3 primary nucleofection solution (V4XP-3024, Lonza). The cells were then added to the Cas9-gRNAs complex. The mixture was nucleofected using the CM-137 condition on a 4D-Nucleofector (4D-Nucleofector Core Unit, AAF-1002B; 4D-Nucleofector X Unit, AAF-1002X, Lonza). The resulting cells were maintained in the presence of M-CSF, and KO efficiency was assessed by flow cytometry.

### RT-quantitative PCR

Gene mRNA expression was determined by qPCR using the CFX Connect Real-Time PCR Detection System (Bio-Rad Laboratories) following reverse transcription with SuperScript Reverse Transcriptase (18064022, Thermo Fisher Scientific). The primers were, *Nos2* forward, 5’-GGCAGCCTGTGAGACCTTTG, reverse, 5’-CGTTTCGGGATCTGAATGTGA (32); *Actb* forward, 5’-GACGGCCAGGTCATCACTATTG, reverse, 5’-AGGAAGGCTGGAAAAGAGACC (33); *Cd44* forward, 5’-TCTGCCATCTAGCACTAAGAGC, reverse, 5’-GTCTGGGTATTGAAAGGTGTAGC; *Il6* forward, 5’-GAGGATACCACTCCCAACAGAC, reverse, 5’-AGCTATGGTACTCCAGAAGACC; *Il1b* forward, 5’-AACCTTTGACCTGGGCTGTC, reverse, 5’-AATGGGAACGTCACACACCA, and *Tnfa* forward, 5’-AGGCACTCCCCCAAAAGATG, reverse, 5’-TTTGCTACGACGTGGGCTAC. Data were normalized to a reference gene *Actb*.

### Statistical analysis

Data, expressed as mean ± SD, were examined for normality (Shapiro-Wilk test) to determine whether to use parametric or nonparametric testing. Differences between groups were calculated for statistical significance using an unpaired Student’s t test for 2 groups or an ANOVA test for 3 or more groups (parametric) or a Mann-Whitney-Wilcoxon test (non-parametric). *P* ≤ 0.05 was considered statistically significant.

## Results

### Environmental materials damage the plasma membrane of macrophages

Macrophages are the most abundant phagocytes residing in the skin, airway, and gut, playing a crucial role in host defense and tissue homeostasis. As foreign materials, environmental materials can activate macrophages and initiate inflammatory responses. However, it is unclear whether such exposures affect macrophage function and impair host defense. To investigate the effects of environmental materials on macrophages, we first examined macrophage cellular membrane integrity following exposure to several environmental substances. Briefly, bone marrow-derived macrophages (BMDMs) were treated with various environmental agents or the vehicle control. The cells were then stained with Annexin V (binding to intramembrane lipids to detect membrane damage) and propidium iodide (PI, staining DNA in apoptotic/necrotic cells). Detergents are broadly used to lyse cells in biological research. As expected, treatment with detergent or another surfactant saponin damaged macrophage membranes compared to the vehicle treatment (**Figure 1A**). Treatments with nanoparticles (carbon nanotubes, WC, and kaolin) also disrupted macrophage membranes (**Figure 1B**, **Supplementary Figure 4**). Additionally, the treatments slightly increased cell death, as revealed by PI staining (**Figure 1**, **Supplementary Figure 4**). These findings suggest that the tested environmental materials can compromise macrophage membranes, and therefore, may influence macrophage function.

**Figure 1:**
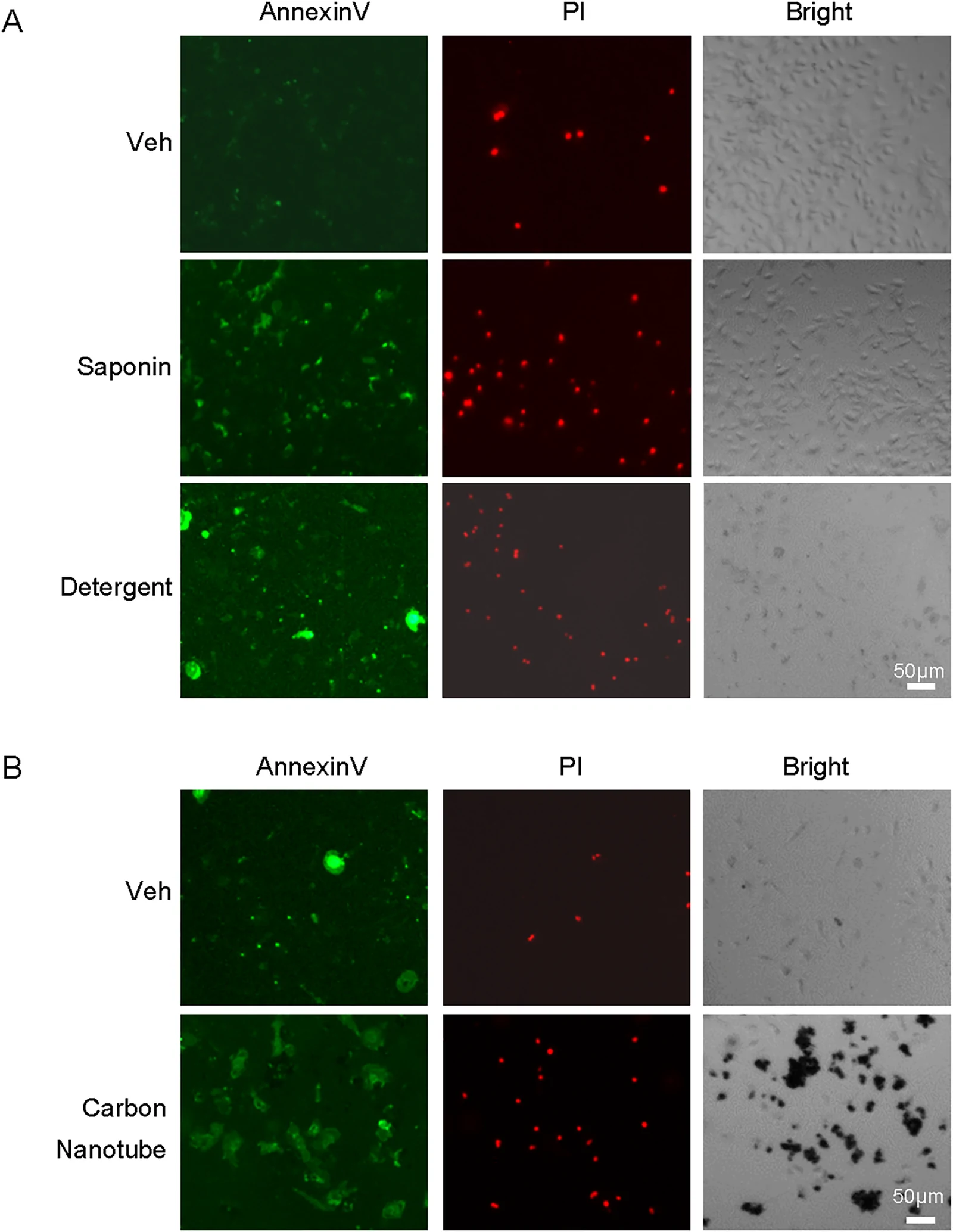
Environmental materials damage macrophage plasma membranes. **(A)** BMDMs were treated with saponin, detergent, or the vehicle. **(B)** BMDMs were treated with carbon nanotubes or the vehicle. Plasma membrane damage was assessed by Annexin V staining, and cell death was indicated by PI staining. Horizontal scale bar, 50 μm. Data shown are representative of 2 independent experiments.

### Membrane damage by environmental materials promotes macrophage fusion

MGCs, a subset of functionally distinct multinucleate giant cells fused from macrophages, play critical roles in tissue homeostasis, host defense, and inflammatory responses. In situations such as granulomatous diseases or implant site reactions, MGCs contribute to chronic inflammatory responses. Macrophage fusion is governed by various signaling pathways, including cytokine-driven mechanisms (18–20, 34, 35). Because the membrane damage is induced by environmental factors but not infections, we used a well-described condition that induces the formation of FBGCs by cytokines M-CSF and IL-4 (28). Early studies showed that needle-shaped nanoparticles promote MGC formation in the lung and tumor (36, 37). As expected, treatment of BMDMs with carbon nanotubes enhanced the formation of FBGCs relative to vehicle treatment when IL-4 and M-CSF were supplied to the culture (**Figure 2A**). Interestingly, treatment with the surfactants saponin and detergent, both of which damage macrophage membranes as carbon nanotubes (**Figure 1**), also promoted the formation of FBGCs (**Figures 2B, C**). These data suggest that membrane damage enhances macrophage fusion, a property of macrophages to respond to foreign materials.

**Figure 2:**
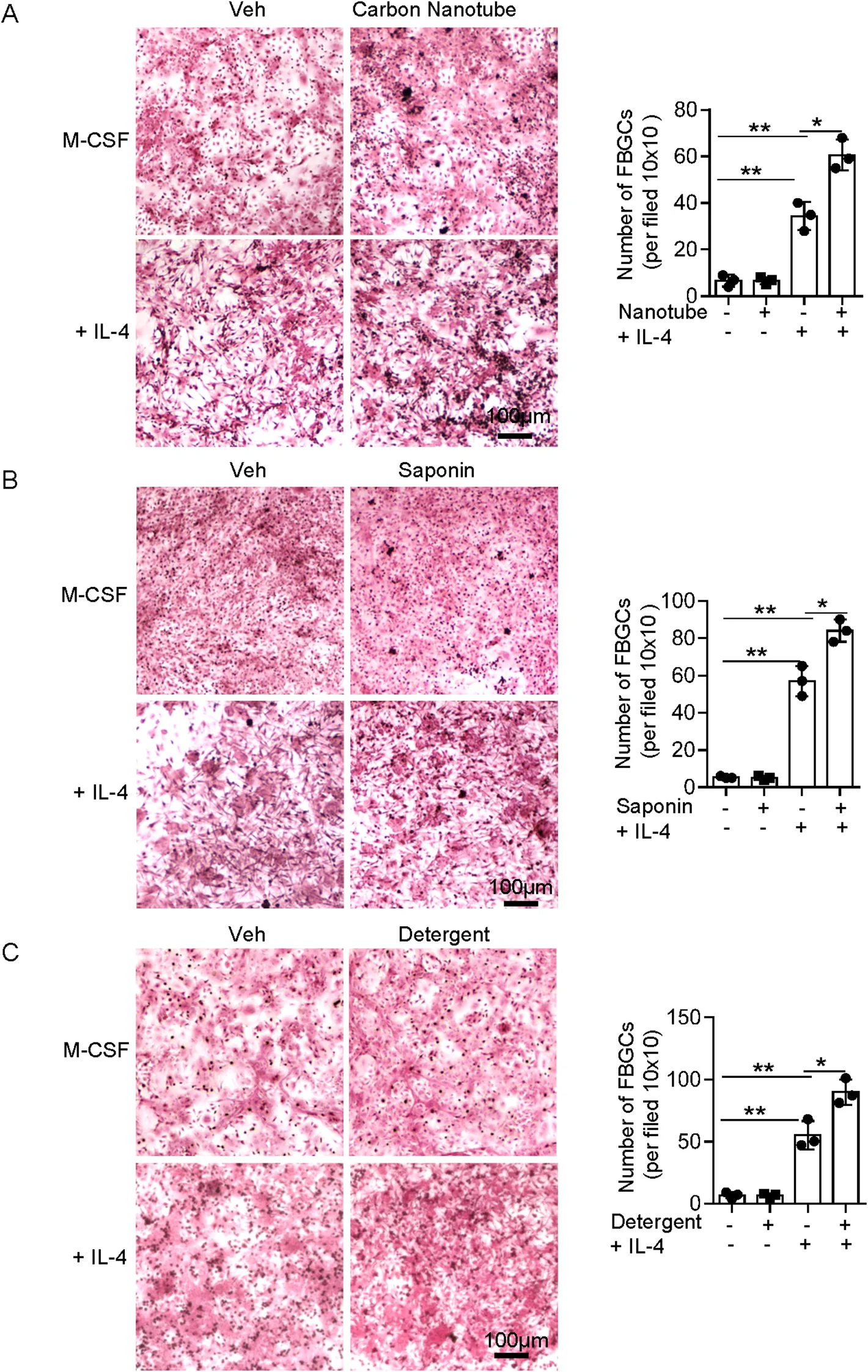
Environmental material-caused membrane damage promotes macrophage fusion. BMDMs were treated with carbon nanotubes **(A)**, saponin **(B)**, detergent **(C)**, or the vehicle and then cultured with or without IL-4. Macrophage fusion was assessed by H&E staining. Horizontal scale bar, 100 μm. FBGC, foreign body giant cells; Veh, vehicle. Data (mean ± SD) shown are representative of 2 independent experiments [n = 3 biological replicates (and hereafter) per group]. ANOVA test, *p < 0.05; **p < 0.005.

### Saponin-induced macrophage fusion is CD44-dependent

CD44, a cell surface adhesive molecule essential in cell-cell interactions, migration, and adhesion, plays a vital role in macrophage fusion (38–40). Because environmental materials interact with and damage cellular membranes, we inferred that these materials interfere with CD44 function and even its expression on macrophage surfaces. To test this, we treated macrophages with saponin and observed elevated CD44 levels by flow cytometry followed by surface staining of CD44 (**Figure 3A**). The effects of saponin on CD44 expression were post-transcriptional but not transcriptional (**Supplementary Figure 5**). Next, to explore the role of CD44 in macrophage fusion induced by environmental damage, we successfully generated *Cd44* knockout (KO) BMDMs using a CRISPR-Cas9 system (**Supplementary Figure 6**) and treated the cells with or without IL-4 in the presence of M-CSF. As shown in **Figure 2**, without the addition of IL-4, we observed only a few cell-cell fusions in either *Cd44* WT or KO BMDMs in the presence or absence of saponin (**Supplementary Figure 7**). Consistently, administration of IL-4 promoted MGC formation in WT BMDMs, whereas addition of saponin further enhanced this process, displaying greater numbers of MGCs, of which more cells contained 3 or more nuclei (**Figures 3B, C**). Under these conditions, cell-cell fusion in *Cd44* KO BMDMs was significantly decreased, and the effects of saponin on macrophage fusion were diminished (**Figures 3B, C**), suggesting that saponin-mediated enhancement of macrophage fusion is CD44-dependent.

**Figure 3:**
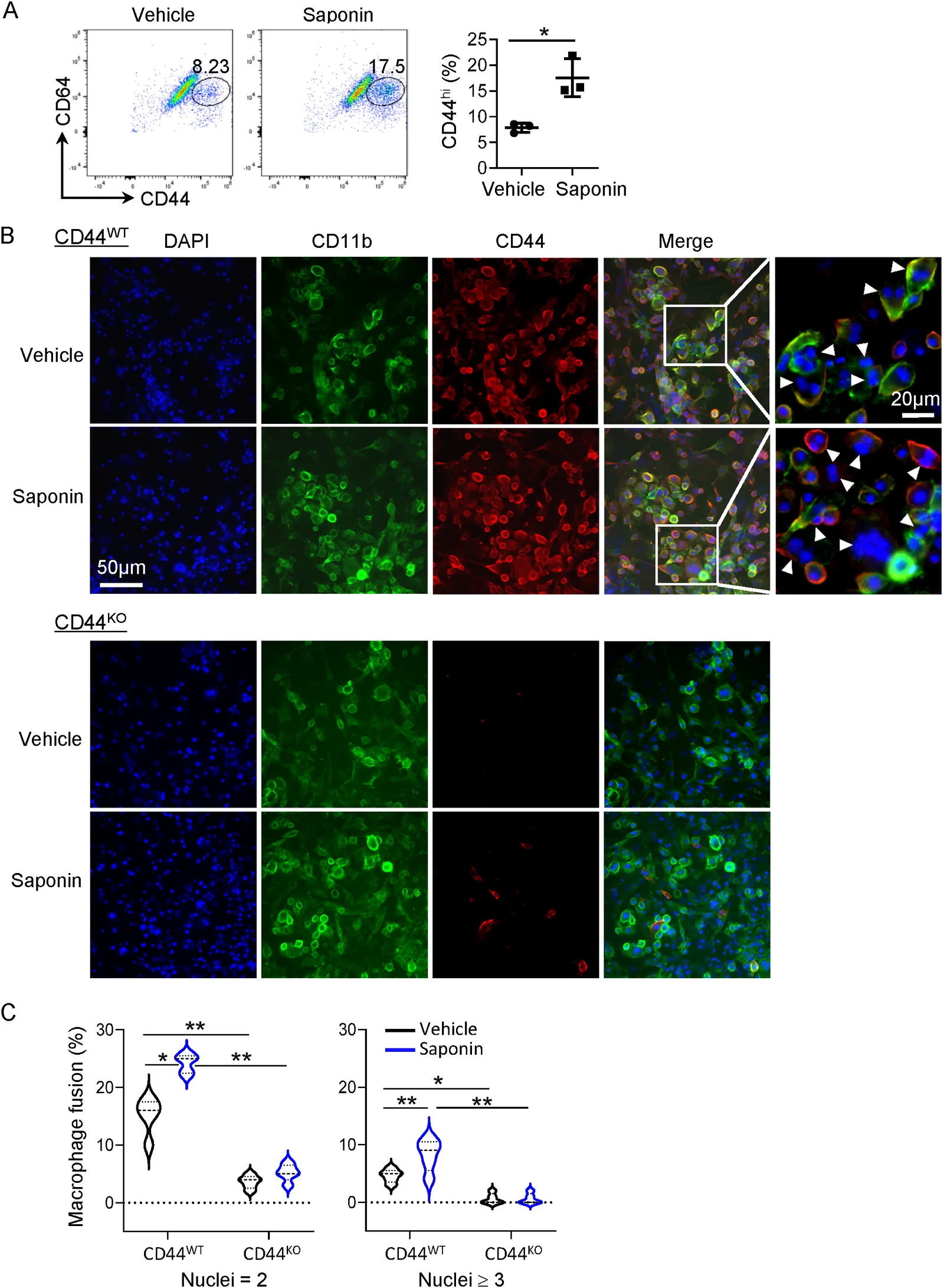
Saponin enhances macrophage fusion via upregulation of CD44. **(A)** Flow cytometry of CD44 expression on BMDMs after treatment with or without saponin. **(B)** Representative fluorescent images of *Cd44*-WT (Upper) or *Cd44*-deficient (Lower) BMDMs treated with saponin or the vehicle in the presence of IL-4. Images were acquired by using Cellomics. MGCs are indicated by arrowheads. **(C)** Statistical analysis of macrophage fusion in **(B)**. One representative image per sample (n = 5) was assessed. Percentages of cells with 2 or ≥3 nuclei were depicted. Data (mean ± SD) shown are representative of 3 independent experiments. Student’s *t*-test **(A)** or ANOVA test **(C)**, *p < 0.05.

### Saponin and detergent inhibit the inflammatory function of macrophages

ROS, one of the most important antimicrobial mediators produced by macrophages, serves as a major defense mechanism against pathogens. To understand how environmental material-induced membrane damage affects macrophage defensive function, we investigated ROS production in macrophages in response to these materials. BMDMs were treated with saponin, detergent, or the vehicle, and levels of ROS were assessed by DCFH-DA staining. Both saponin and detergent significantly decreased ROS levels compared to the vehicle (**Figures 4A, B. D, E**). As revealed by reverse transcription-polymerase chain reaction (RT-qPCR), treatment with saponin or detergent suppressed mRNA expression of macrophage-associated markers, such as pro-inflammatory cytokines (IL-1β, IL-6, and TNFα) and iNOS (encoded by *Nos2*) relative to treatment with the vehicle (**Figures 4C–F**). These results indicate that exposure to environmental materials inhibits the expression of inflammatory mediators in macrophages, manifesting the negative consequence of damage to plasma membranes, as the membranes assemble receptors, adaptor proteins, and kinases critical for signal transduction.

**Figure 4:**
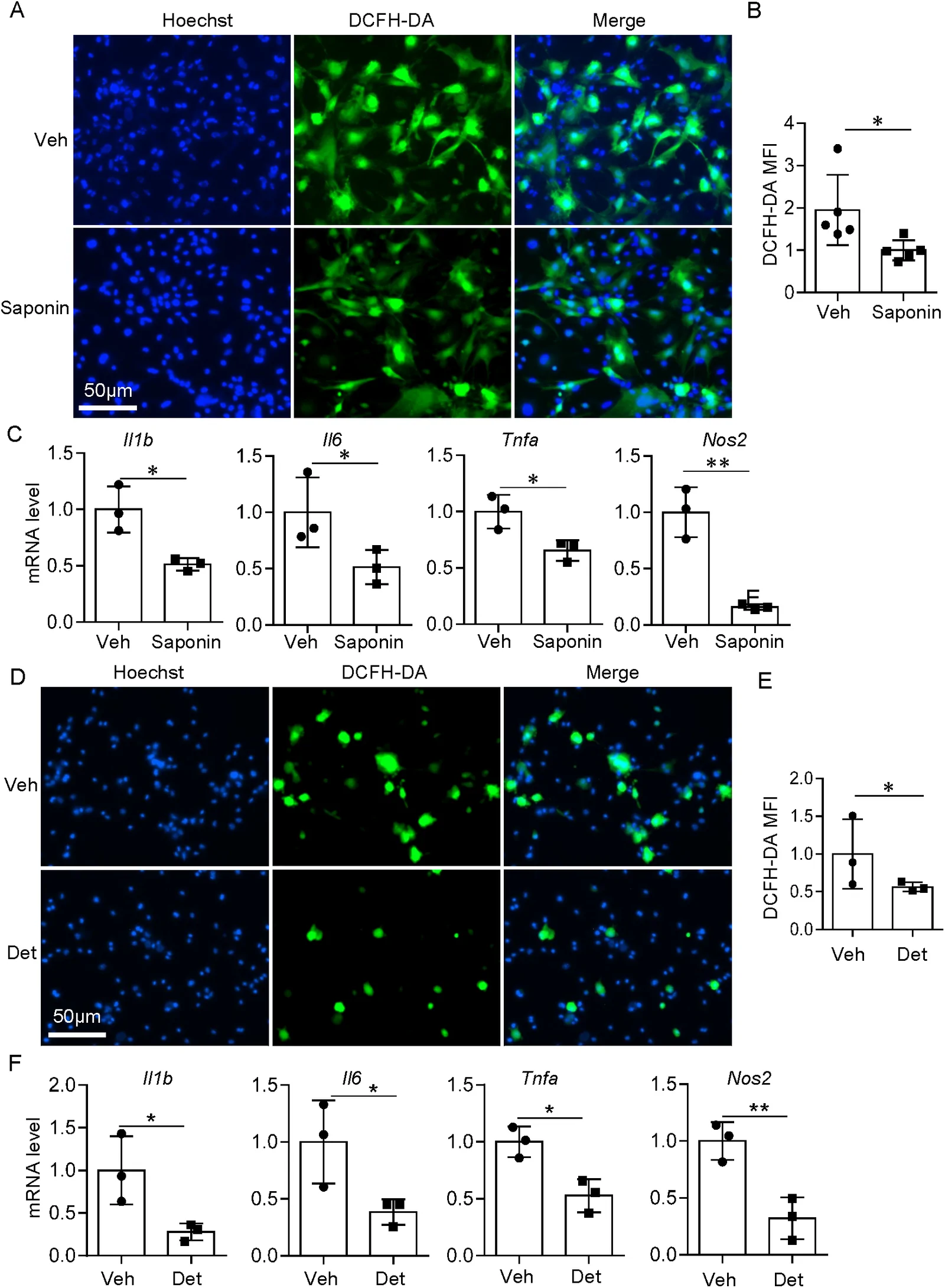
Environmental materials (saponin and detergent) decrease the production of ROS and pro-inflammatory cytokines in macrophages. BMDMs were treated with saponin **(A-C)**, detergent **(D-F)**, or the vehicle and stained with DCFH-DA and Hoechst. Veh, vehicle; Det, detergent. **(A, D)** Representative fluorescent images. Horizontal scale bar, 50 μm. **(B, E)** Mean fluorescent intensity (MFI) of DCFH-DA. **(C, F)** RT-qPCR of mRNA expression of indicated genes. *Actb* was used as an internal loading control. Data (mean ± SD) shown are representative of 2 independent experiments (n = 3 per group). Student’s *t*-test, *p < 0.05; **p < 0.005.

### Damage to macrophage membranes decreases macrophage phagocytosis

Macrophages are key players in the immune system through detecting and clearing pathogens through phagocytosis. This process is vital for host defense against a wide range of pathogens, including fungi and bacteria. To further understand how membrane damage influences macrophage function, we treated BMDMs with saponin for 10 minutes, then washed the cells and incubated them with CFSE-labeled live *E. coli* for 30 minutes. Following fixation, the cells were stained with DAPI to visualize macrophage nuclei. We found that treatment with saponin significantly decreased phagocytosis in macrophages compared to the vehicle-treated cells (**Figures 5A, B**). We also assessed the impact of laundry detergent on macrophage phagocytosis. BMDMs were treated with detergent for 10 minutes, and after being washed, co-cultured with CFSE-labeled live *C. albicans* for 30 minutes. Similar to phagocytosis of *E. coli*, treatment with detergent impaired phagocytosis of *C. albicans* in macrophages compared to the vehicle-treated cells (**Figures 5C, D**). These results indicate that both detergent and saponin impair macrophage phagocytosis, which may be due to the membrane damage caused by the environmental factors. Our findings suggest that environmental exposures may impair immune functions and thus increase susceptibility to infections.

**Figure 5:**
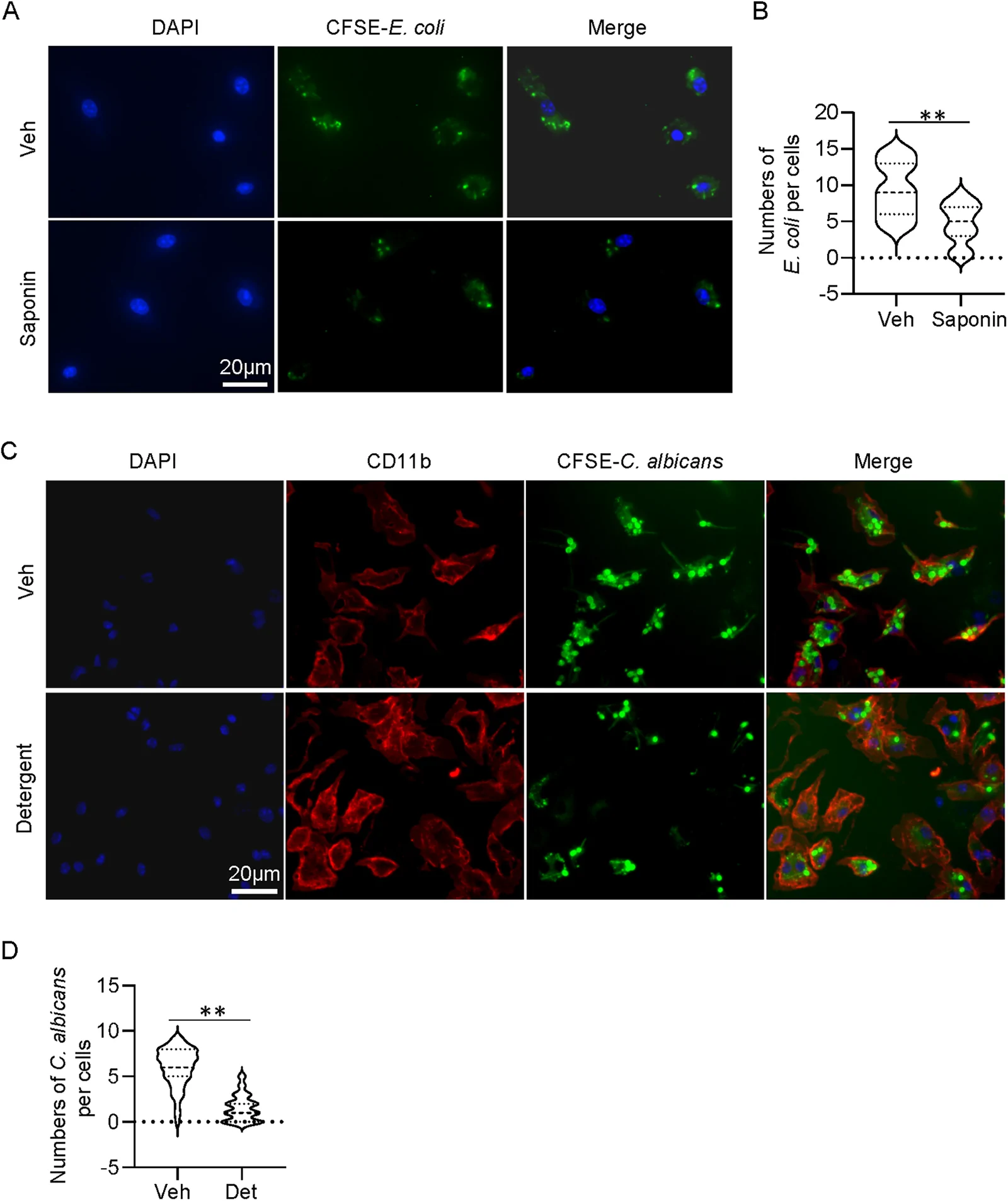
Environmental material-caused membrane damage impairs the phagocytic function of macrophages. BMDMs were cocultured with CFSE-labelled *E. coli* **(A, B)** or *C. albicans* **(C, D)**. After fixation, the cells were stained with CD11b (red) and DAPI (blue). **(A, C)** Phagocytosis of *E. coli* **(A)** or *C. albicans* **(C)** by BMDMs treated with/without detergent or saponin, respectively. Horizontal scale bar, 20 μm. **(B, D)** Statistical analysis of **(A, C)**. One hundred cells (n = 100) were assessed in each sample. Det, detergent. Data shown are representative of 3 independent experiments. Student’s *t*-test, **p < 0.005.

### Human THP-1-derived macrophages respond to detergent similarly to mouse macrophages

Human monocytic THP-1 cells were differentiated into macrophage-like cells. When treated with detergent, these cells lost membrane integrity (**Figure 6A**). Detergent also decreased ROS expression and impaired phagocytosis of Candida by THP-1-derived macrophages (**Figures 6B, C**). Thus, human THP-1-derived macrophages behave similarly to murine macrophages in response to detergent, and may also be affected by other environmental materials.

**Figure 6:**
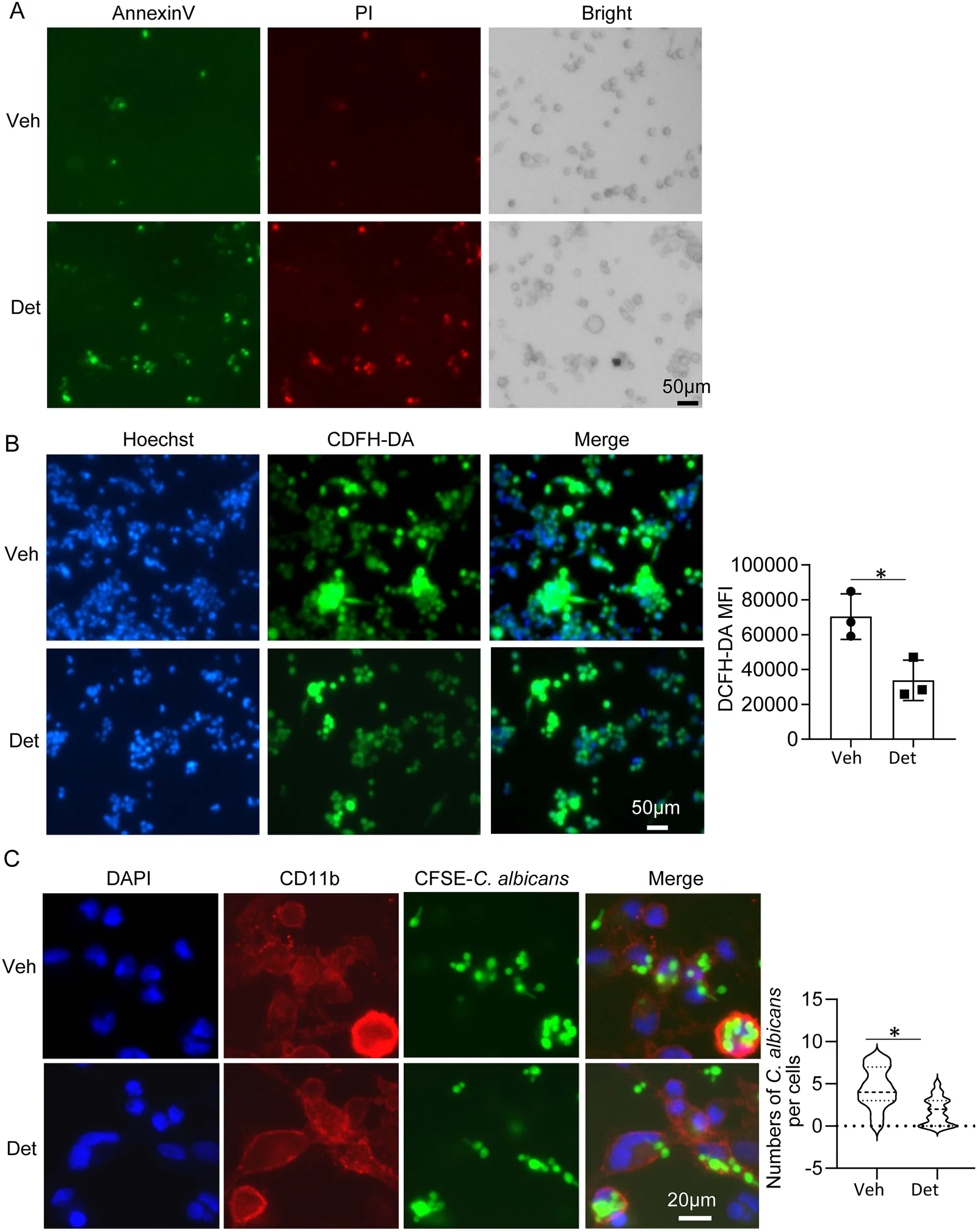
Detergent damages human macrophage plasma membranes and impairs their ROS expression and phagocytic function. Human macrophages, differentiated from THP-1 cells, were treated with detergent or the vehicle. **(A)** Plasma membrane damage was assessed by Annexin V staining, and cell death was indicated by PI staining. Horizontal scale bar, 50 μm. **(B)** ROS expression was assessed by DCFH-DA staining and Hoechst was used to reveal nuclei. Horizontal scale bar, 50 μm. **(C)** Phagocytosis of *C. albicans* by THP-1-differentiated macrophages treated with detergent or vehicle. Horizontal scale bar, 20 μm. One hundred cells (n = 100) were assessed in each sample. Veh, vehicle; Det, detergent. Data shown are representative of 3 independent experiments. Student’s *t*-test, *p < 0.05.

### Airway exposure to laundry detergent increases susceptibility to Candida infection in mice

Laundry detergents, commonly used for cleaning clothes and linens, are known to cause respiratory complications, including asthma (9–12). To understand the effects of environmental hazards on host defense, we examined the effects of laundry detergents using a mouse model of Candida airway infection. Mice were intratracheally (i.t.) administered a laundry detergent or PBS, followed by a Candida i.t. challenge on the same day, once daily for four consecutive days. The mice were analyzed the next day after the last dose of Candida (**Figure 7A**). Using colony-forming unit (CFU) assays, we found that the detergent-treated group had higher fungal burdens and lower body weights than the control group (**Figures 7B, C**). Administration of detergent decreased the numbers of macrophages, eosinophils, and neutrophils in the BALF (**Figure 7D**), suggesting that exposure to detergent impaired innate immune responses in the airway. In addition to innate immune profiles, exposure to detergent also impeded adaptive immune responses, including decreases in the frequencies of TH17 (IL-17^+^) and TH1 (IFNγ^+^) cells in the CD4^+^ fraction of BALF cells, and the levels of TH17 cytokine IL-17 and TH1 cytokine IFNγ in BALF (**Figures 7E, F**). To understand how airway exposure to detergent influences T cell responses, we assessed the expression of CD86 and MHC-II by macrophages and dendritic cells, which are primarily involved in costimulation and antigen presentation for T cell activation and expansion. Interestingly, treatment with detergent not only damaged macrophage membranes (**Figure 1B**) but also inhibited CD86 and MHC-II expression in macrophages (**Figures 7G, H**). Similarly, exposure to detergent suppressed CD86 and MHC-II expression in dendritic cells (DCs) (**Figures 7I, J**). Due to impaired adaptive immunity and increased Candida burden, mice receiving detergent had increased accumulation of immune infiltrates in the peribronchovascular spaces compared with the control mice (**Figure 7K**). This may also explain the decreases in recruitment of the myeloid populations into the airway (**Figure 7D**), where they directly interact with and clear the pathogens. Decreases in CD86 and MHC-II expression in DCs and macrophages were also observed in mice receiving detergent alone (**Supplementary Figure 8**), suggesting that exposure to detergent directly suppresses antigen-presenting cell function. Interestingly, detergent treatment increased myeloid cell infiltration into the airway (**Supplementary Figure 8**). This may result from detergent-induced damage to the airway epithelial barrier. In the presence of Candida, the pathogenic signals recruit more myeloid cells, which overwhelm those induced by detergent. Collectively, treatment with laundry detergent suppresses the antigen presentation and costimulatory function of macrophages and DCs, leading to decreased immune responses in both innate and adaptive arms of the immune system, subsequently hindering host defense.

**Figure 7:**
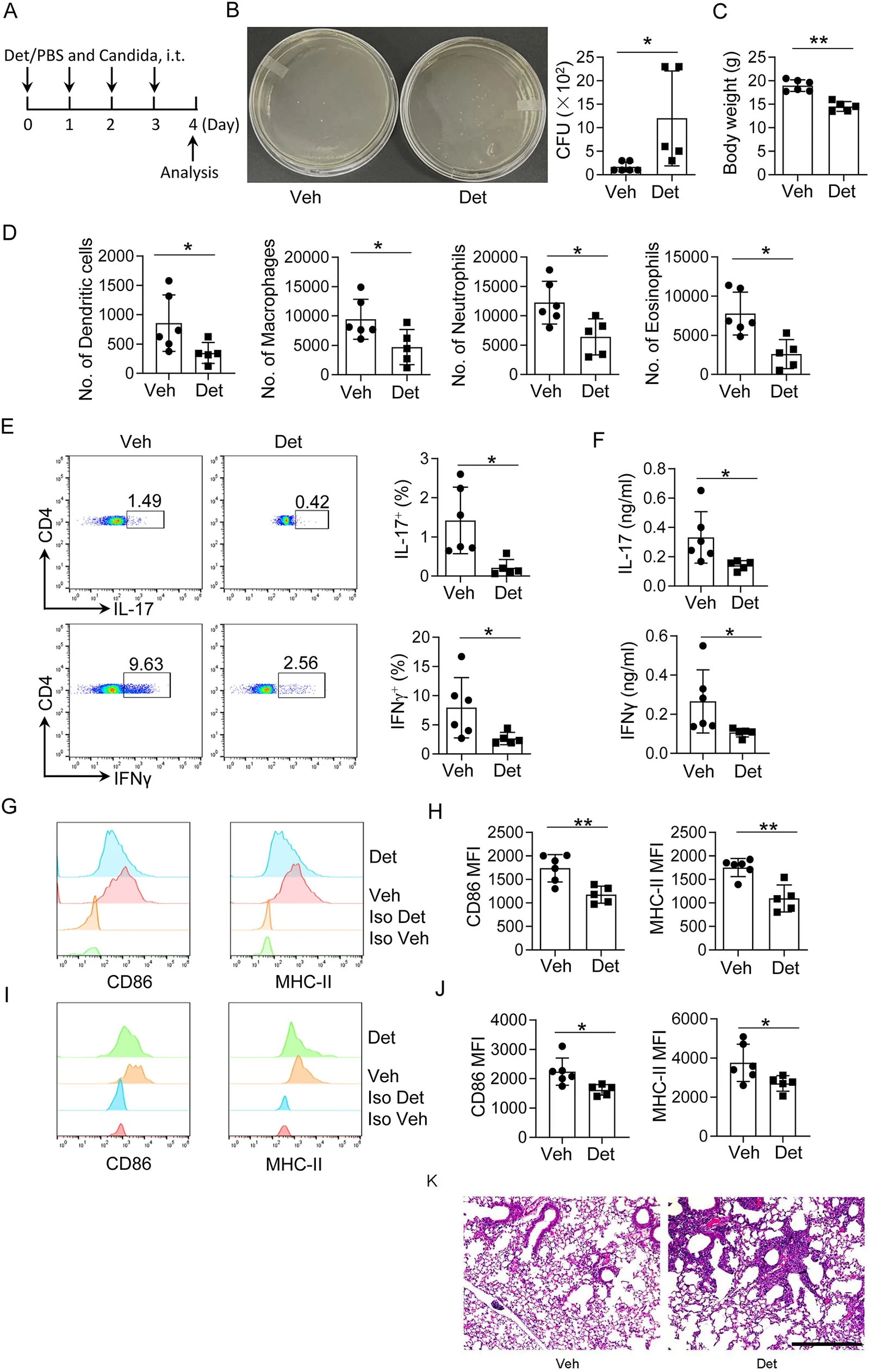
Laundry detergent increases susceptibility to Candida airway infection in mice. **(A)** C57BL/6 mice were intratracheally (i.t.) administered live Candida after i.t. receiving a laundry detergent or PBS once daily for four consecutive days, and sacrificed the next day after the last dose of Candida. **(B)** Colony-forming unit (CFU) assays of Candida burden in the bronchoalveolar lavage fluids (BALF). **(C)** Body weights of the two groups of mice. **(D)** Profiles of myeloid cells in BALF. **(E)** Flow cytometry of IL-17- and IFNγ-expressing CD4^+^ TH cells in BALF. **(F)** ELISA of IL-17 and IFNγ in BALF. **(G)** Flow cytometry of MHC-II and CD86 on macrophages on a CD11b^+^Cd11c^lo^ gate. **(H)** Mean fluorescence intensity of MHC-II and CD86 in **(G, I)** Flow cytometry of MHC-II and CD86 on dendritic cells on a CD11b^+^Cd11c^hi^ gate. **(J)** Mean fluorescence intensity of MHC-II and CD86 in **(I, K)** H&E staining of lung tissues. Horizontal scale bar, 400 μm. Veh, vehicle; Det, detergent; Iso, isotype control. Data (mean ± SD) are representative of two experiments. n = 5–6 per group. Student’s *t*-test, *p < 0.05; **p < 0.005.

## Discussion

Macrophages, a critical compartment of the innate immune system, also bridge innate and adaptive immunity through recognition, phagocytosis, and digestion of pathogens and foreign materials, and presentation of antigens to T cells, mediating T cell activation and expansion. While environmental materials, such as tungsten carbide, nanotubes, and detergents, are known to influence macrophage function (16, 17, 41), the underlying mechanisms are not fully understood.

Cell membranes serve as a barrier separating the cell’s inner contents from its external environment and are responsible for cell-cell and cell-environment communications. Intensive studies have shown that various mechanisms, including interaction with environmental materials, excessive immune responses, and cell ageing, can cause cell membrane damage, leading to alterations in cell function and even cell death (42, 43). Because macrophages reside in the alveolar and intraepithelial spaces of the lung, as well as the skin and gut, where they have the opportunity to interact with the environment, we hypothesize that environmental materials cause membrane damage and subsequently impair cell function. Indeed, our study has found that nanoparticles (WC, carbon nanotubes, and kaolin) and soluble surfactants (saponin and laundry detergent) can induce cell membrane damage in macrophages, but during the tested period, only slightly increase cell death (**Figure 1**). Therefore, many environmental factors can disrupt the integrity of the plasma membrane of macrophages and thus may lead to negative consequences for cell function.

MGCs, formed from macrophage fusion, have unique functions in the immune system. In tuberculosis, MGCs form granulomas that constrain Mycobacterium tuberculosis when the bacteria escape from lysosomes by preventing phagosome-lysosome fusion and can survive within the host cells (44). This is considered a defense mechanism to mitigate host tissue damage. Similarly, in the lungs of hard metal disease (21–25) and silicosis (45), macrophages fuse into MGCs; however, these MGCs fail to clear the ingested hard metal or silica particles, and the accumulation of MGCs results in deadly inflammation and tissue destruction. Whether cell membrane damage contributes to the pathogenesis of MGC diseases is unclear. Since there is a lack of infectious clues in exposure to environmental materials compared to pathogens, we examined the influence of macrophage membrane damage on the formation of FBGCs. Interestingly, both carbon nanotubes and surfactants (saponin and detergent) facilitate MGC formation (**Figure 2**), although the underlying molecular mechanism remains unknown. CD44, a cell surface adhesive molecule, plays a vital role in macrophage fusion (38–40). We observed that saponin, one of the tested environmental hazards, upregulates CD44 expression on macrophages; KO of *Cd44* diminishes the stimulating effects of saponin on macrophage fusion (**Figures 3**, **4**). In summary, environmental factors damage macrophage membranes and elevate CD44 expression; together, these alterations promote MGC formation. This mechanistic insight may also hold true for other multinucleated GCs, such as LGCs and osteoclasts, and suggest therapeutic targets.

Since macrophages play a pathogenic role in many diseases, such as rheumatoid arthritis, atherosclerosis, and acute respiratory distress syndrome, targeting macrophages emerges as an attractive strategy. Nanoparticles or drug-bearing nanoparticles are frequently used to deplete M1 macrophages or repolarize M1 macrophages into a less inflammatory M2 phenotype (46). However, whether these nanoparticles crosstalk with environmental toxins, leading to confounding consequences in macrophages, and whether they indirectly affect adaptive immunity need further studies.

As a defense mechanism, macrophages recognize the pathogen-associated molecular patterns (PAMPs) carried by pathogens through pattern recognition receptors (PRRs), such as Toll-like receptors (TLRs) and C-type lectin receptors (CLRs), that mediate the recognition of bacteria and fungi, leading to phagocytosis of these pathogens and formation of a phagosome. The phagosome containing engulfed pathogens then fuses with a lysosome. It forms a phagolysosome, where an array of active chemical materials [including reactive oxygen species (ROS) and nitric oxides] and enzymes digest and clear the phagocytosed pathogens. To determine whether environmental factors disrupt the defense mechanisms of macrophages, we evaluated the effect of surfactants on reactive oxygen species (ROS) expression. Treatment with either saponin or detergent results in a decrease in ROS expression in macrophages (**Figure 4**), suggesting that surfactants suppress lysosomal function in these cells, which is associated with membrane damage caused by environmental materials. Furthermore, surfactants decrease the expression of mRNAs encoding pro-inflammatory cytokines (IL-1β, IL-6, and TNFα) and the inducible nitric oxide synthase (INOS or Nos2) (**Figure 4**), weakening the defensive and inflammatory functions of macrophages. Given that many receptors, kinases, and signal adaptor proteins anchor in or dock inside the plasma membrane, membrane damage contributes to the disturbance of incoming signals. Currently, we cannot rule out whether the surfactants may enter the cells and disrupt cell function via interaction with intracellular signal components. As discussed above, macrophages utilize PRRs to recognize pathogens. Because of this, we inferred that membrane disruption, especially the organization/clustering of pathogen recognition receptors, by environmental materials impairs the phagocytic function of macrophages. Our research confirmed that treatment with both saponin and detergent dramatically decreases the phagocytosis of *E. coli* and *C. albicans*, respectively, by human and mouse macrophages (**Figures 5**, **6**), which is associated with cellular membrane damage in these cells (**Figures 1**, **6**). A limitation here is that THP-1 cell line-derived human macrophages may not fully recapitulate human macrophages. The impact of environmental toxins on human macrophages warrants future studies.

Exposure to various environmental materials through the airway can lead to lung complications. Inhalation of PM, such as carbon nanotubes, can cause airway inflammation and lung damage (7, 8), whereas exposure to detergent residues can result in airway constriction and hyperresponsiveness, increasing the risk of asthma (9, 10). We also found that surfactants inhibit the expression of ROS and decrease the phagocytic function of macrophages (see discussion above). However, it is not clear whether and to what extent these environmental toxins influence host defense. To further evaluate this, we used a Candida airway infection mouse model. Airway exposure to detergent increases fungal burdens in the lung, associated with decreased airway infiltration of myeloid cells, including dendritic cells, macrophages, neutrophils, and eosinophils (**Figures 7B, D**), of which macrophages and neutrophils play essential roles in anti-fungal immunity (47). Attenuation of the NLRP3 inflammasome has been shown to promote M2 macrophage transformation (48), which may impair host defense function. Whether environmental toxins also drive M2 polarization of macrophages is to be determined. Moreover, treatment with detergent also suppresses adaptive immunity, manifesting decreased frequencies of TH1 and TH17 cells and their signature cytokines, IFNγ and IL-17, respectively, in the BALF (**Figures 7E, F**). T cell responses rely on antigen-presenting cells, including DCs and macrophages, to present antigens through their MHC complexes (for TH cells, MHC-II) and to provide costimulation through the B7 molecules. Airway exposure to detergent inhibits the expression of CD86, a member of the B7 family, and MHC-II on macrophages and DCs (**Figures 7G–J**). In line with the decreases in the expression of pro-inflammatory cytokines (IL-1β, IL-6, and TNFα) in macrophages (**Figure 4**), inhibition of CD86 and MHC-II leads to a defect in TH1 and TH17 cell responses. Our study demonstrates that acute exposure to detergent impairs host defense. Whether this phenomenon is reversible needs further investigation. Taken together, airway exposure to detergent results in the decline of both innate and adaptive arms of the immune system, therefore impairing host defense against fungal airway infection.

In conclusion, our study provides evidence that various environmental materials compromise macrophage membrane integrity, impair key immune functions, and promote pathological cell fusion. These alterations contribute to immune dysfunction, leading to increased susceptibility to infection. The findings underscore the need for more rigorous investigation of environmental exposures and warrant the development of strategies to mitigate their immunotoxicity, for example, targeting CD44 or other associated molecules.

## Data Availability

The original contributions presented in the study are included in the article/**Supplementary Material**. Further inquiries can be directed to the corresponding author.
