# Supplementary material for: Plasma membrane damage by environmental materials enhances cell-cell fusion and impairs defense functions of macrophages
Source: Front Immunol. 2026 Sep 16;17:1898611. doi: 10.3389/fimmu.2026.1898611 (PMC13623549; doi:10.3389/fimmu.2026.1898611)
Supplement: Supplementary file 1 [file DataSheet1.pdf]

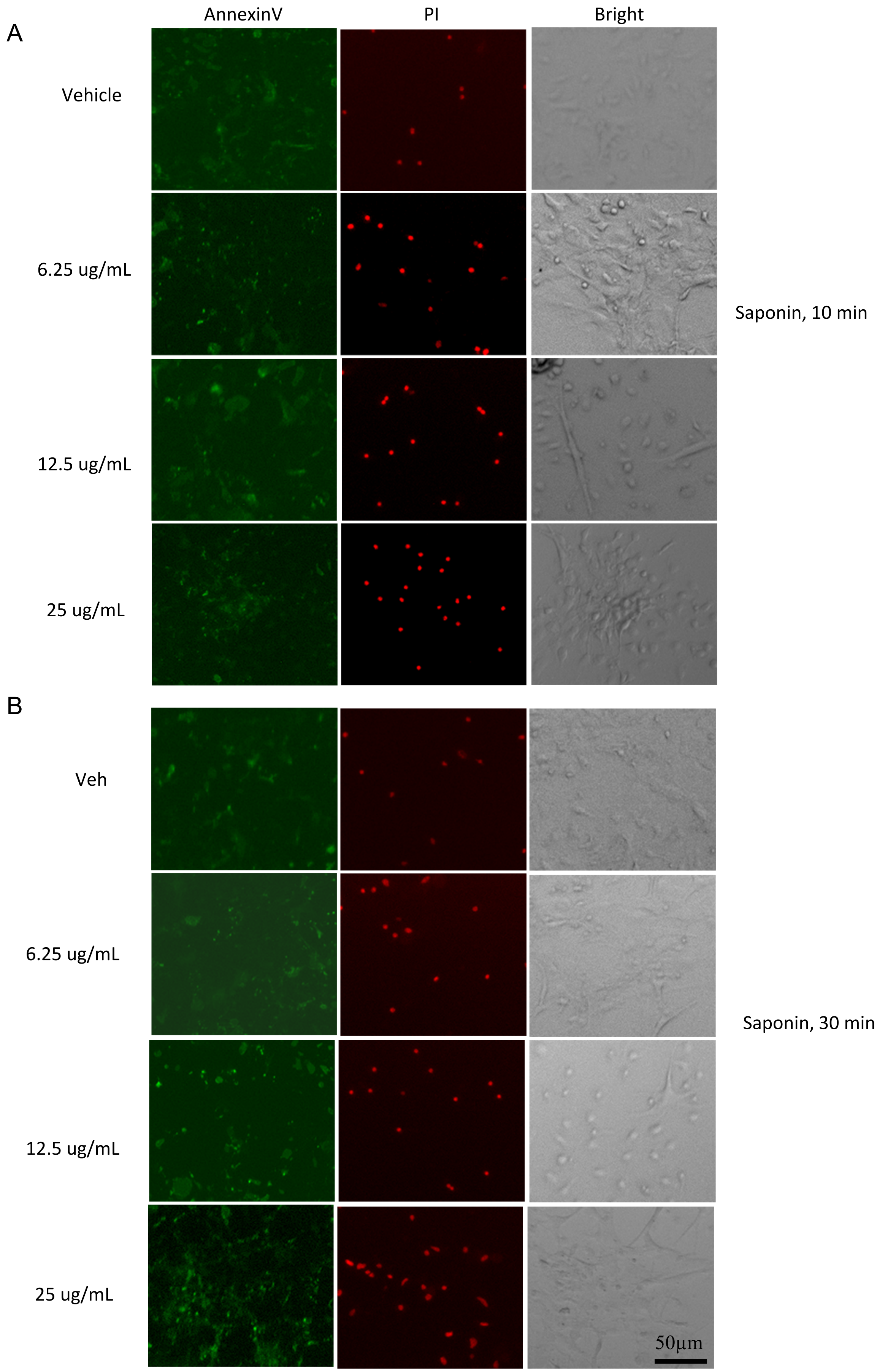

A
AnnexinV
PI
Bright
Vehicle
6.25 ug/mL
12.5 ug/mL
25 ug/mL
Saponin, 10 min
B
Veh
6.25 ug/mL
12.5 ug/mL
25 ug/mL
Saponin, 30 min
50μm

C

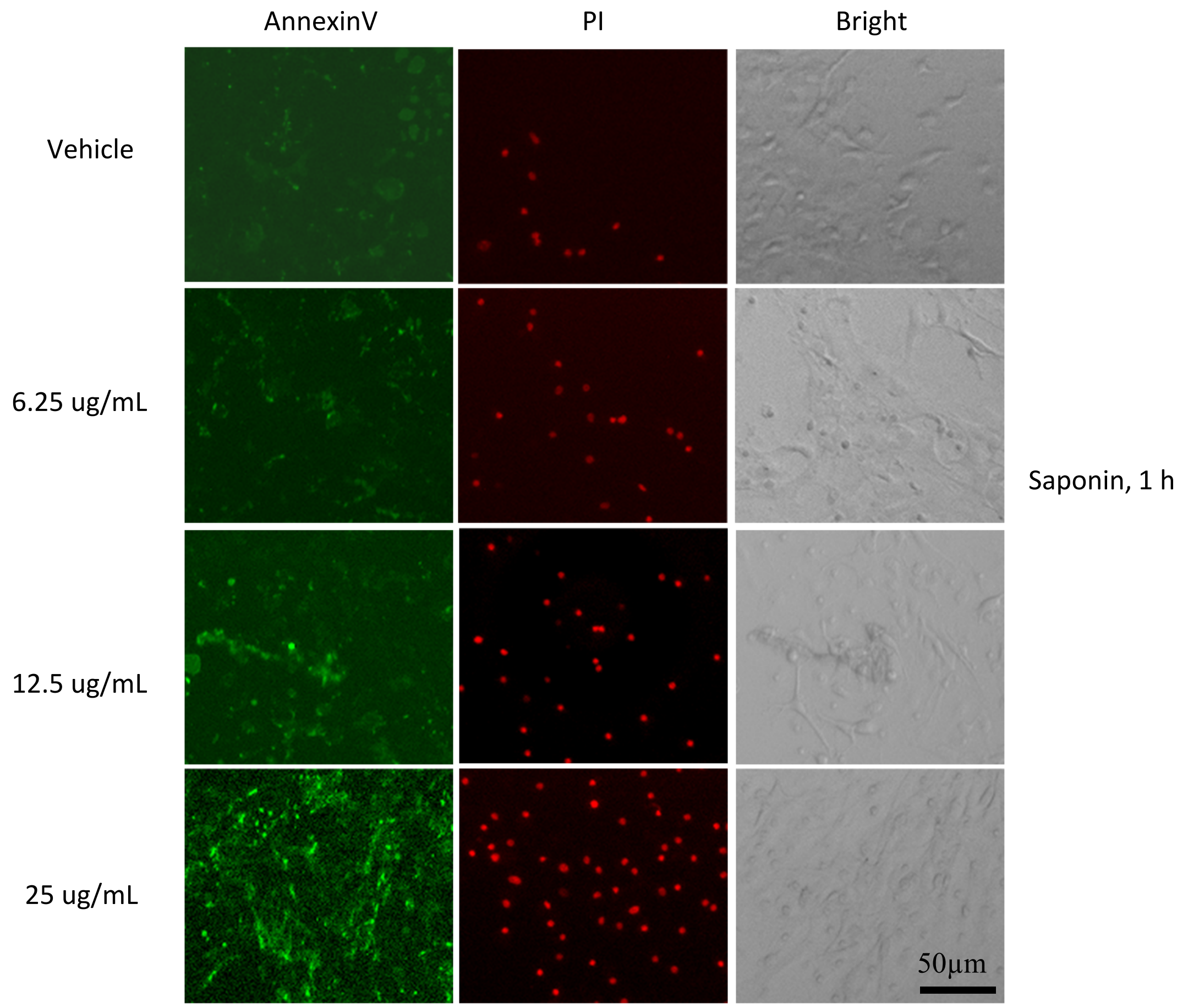

Supplemental Figure 1. The dose-response and time course of saponin-caused damage to macrophage plasma membranes. BMDMs were treated with indicated concentrations of saponin at 10 min (A), 30 min (B), and 1 h (C). Plasma membrane damage was assessed by Annexin V staining, and cell death was indicated by PI staining. Horizontal scale bar, 50 μm. Data shown are representative of 2 independent experiments.

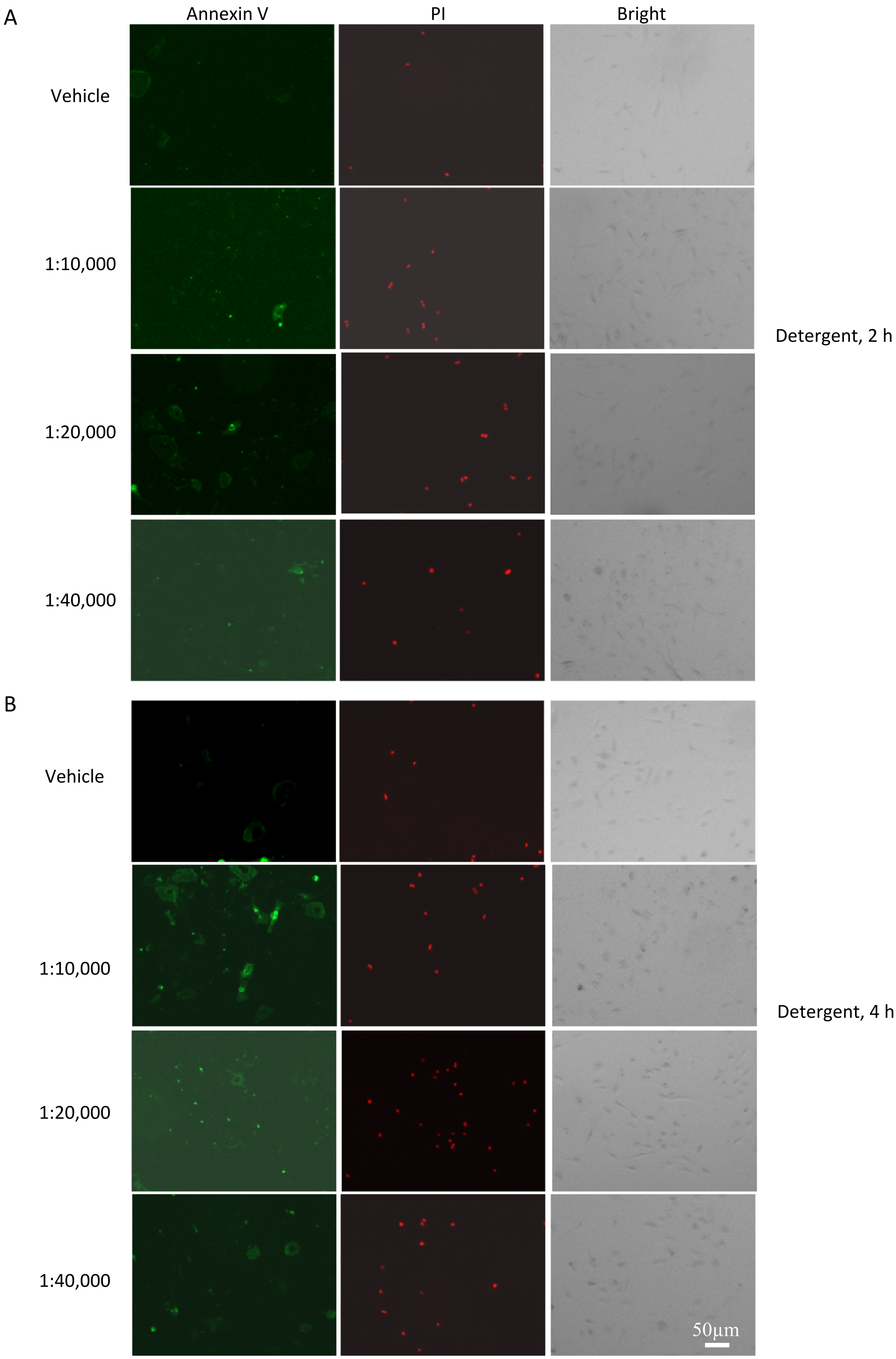
A
Annexin V
PI
Bright
Vehicle
1:10,000
1:20,000
1:40,000
Detergent, 2 h
B
Vehicle
1:10,000
1:20,000
1:40,000
Detergent, 4 h
50μm

Supplemental Figure 2. The dose-response and time course of detergent-caused damage to macrophage plasma membranes. BMDMs were treated with indicated concentrations (dilutions) of laundry detergent at 2 h (A) and 4 h (B). Plasma membrane damage was assessed by Annexin V staining, and cell death was indicated by PI staining. Horizontal scale bar, 50 µm. Data shown are representative of 2 independent experiments.

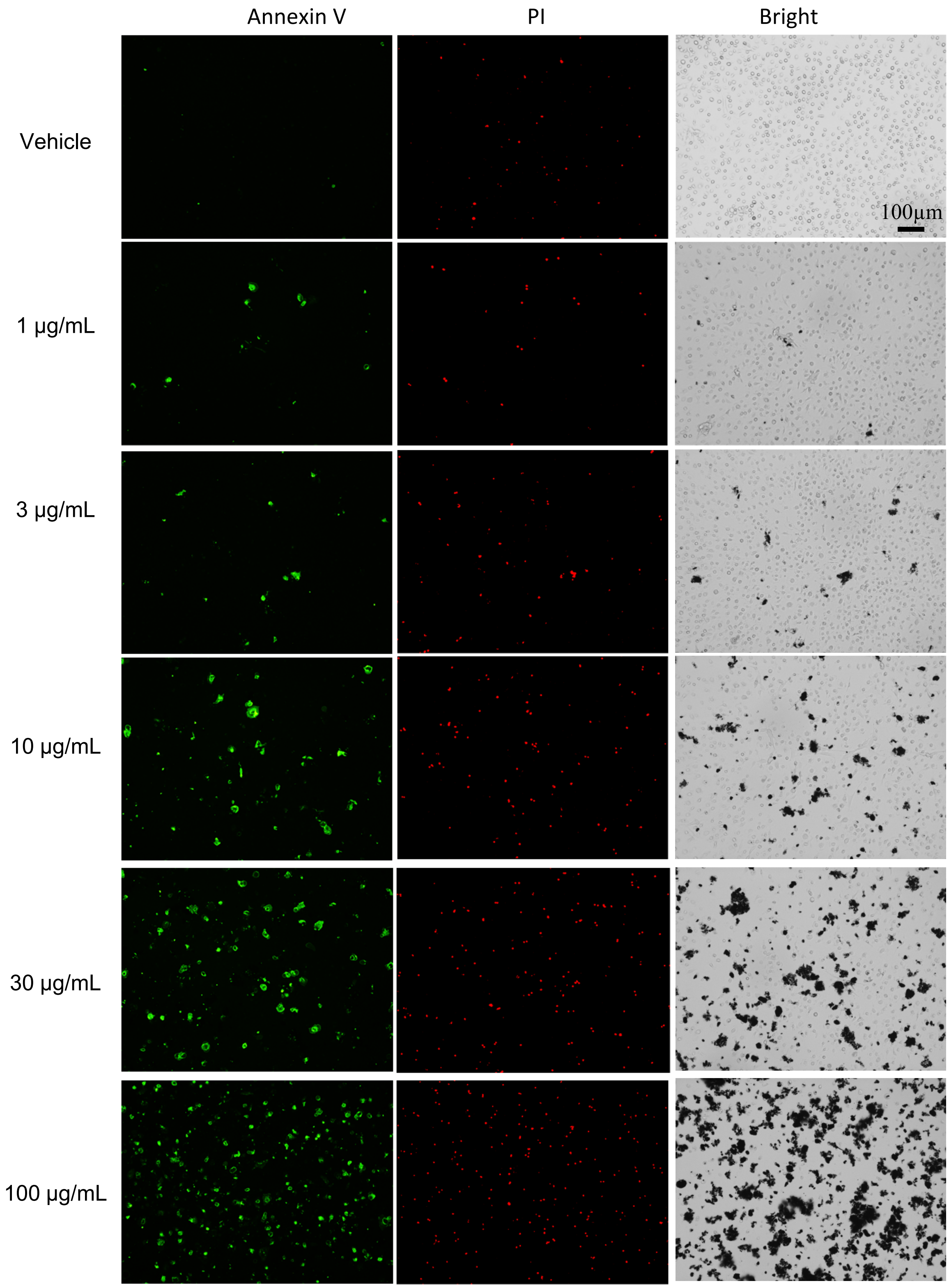

Supplemental Figure 3. The dose-response of carbon nanotube-caused damage to macrophage plasma membranes. BMDMs were treated with indicated concentrations of carbon nanotubes overnight. Plasma membrane damage was assessed by Annexin V staining, and cell death was indicated by PI staining. Horizontal scale bar, 100 μm. Data shown are representative of 2 independent experiments.

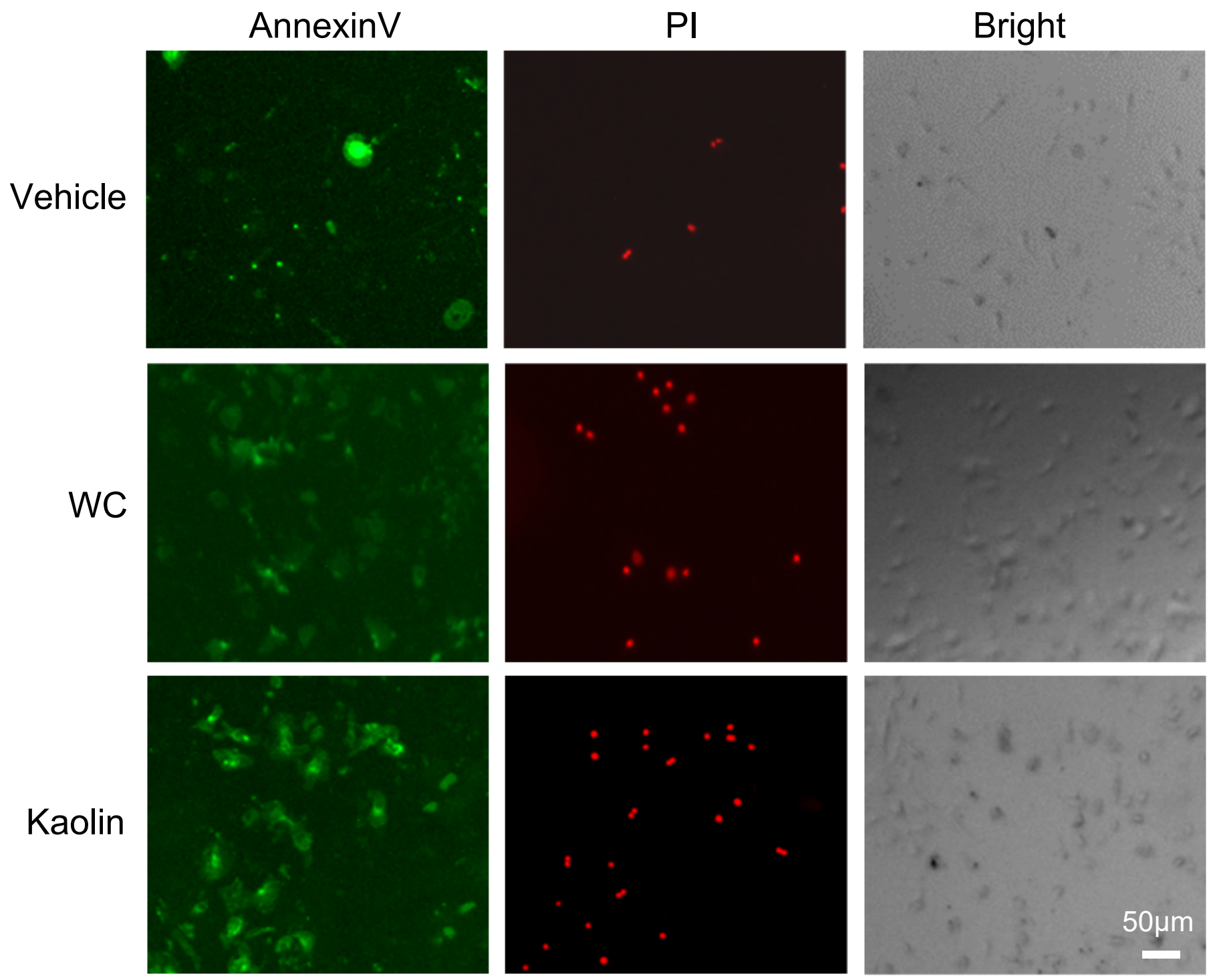

Supplemental Figure 4. Tungsten carbide (WC) and kaolin damage macrophage plasma membranes. BMDMs were treated with 10 µg/mL WC, 10 µg/mL kaolin, or the vehicle overnight. Plasma membrane damage was assessed by Annexin V staining, and cell death was indicated by PI staining. Horizontal scale bar, 50 µm. Data shown are representative of 2 independent experiments. Note: The images of the vehicle control are the same as Figure 1B.

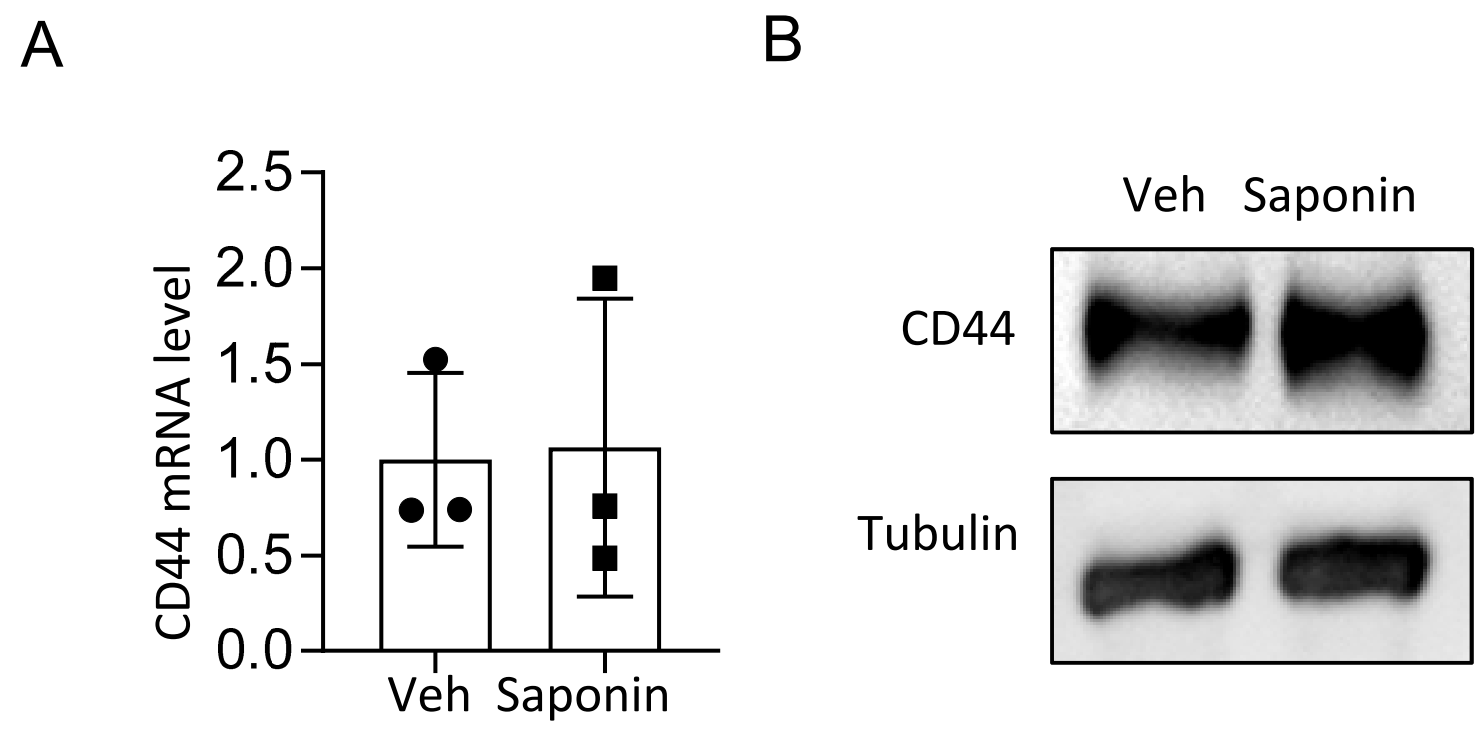

Supplemental Figure 5. Saponin treatment upregulates CD44 expression at the post-transcriptional level. BMDMs were treated with 12.5 μg/mL saponin or the vehicle (Veh) for 10 min. CD44 expression was detected by RT-qPCR at the mRNA level (A) or western blot at the protein level (B). Data shown are representative of 2 independent experiments.

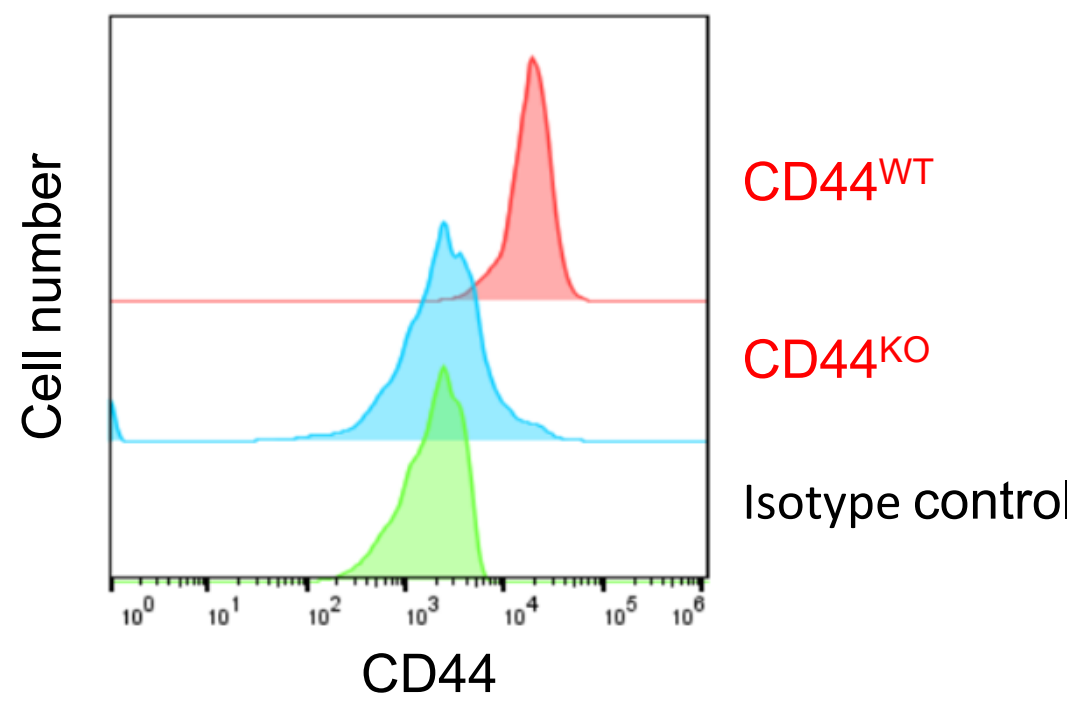

Supplemental Figure 6. CRISPR-Cas9 knockout of *Cd44*. Flow cytometry of CD44 expression on BMDMs with or without CRISPR-Cas9 knockout of *Cd44*. Isotype staining was used as the baseline control. Data shown are representative of 2 independent experiments.

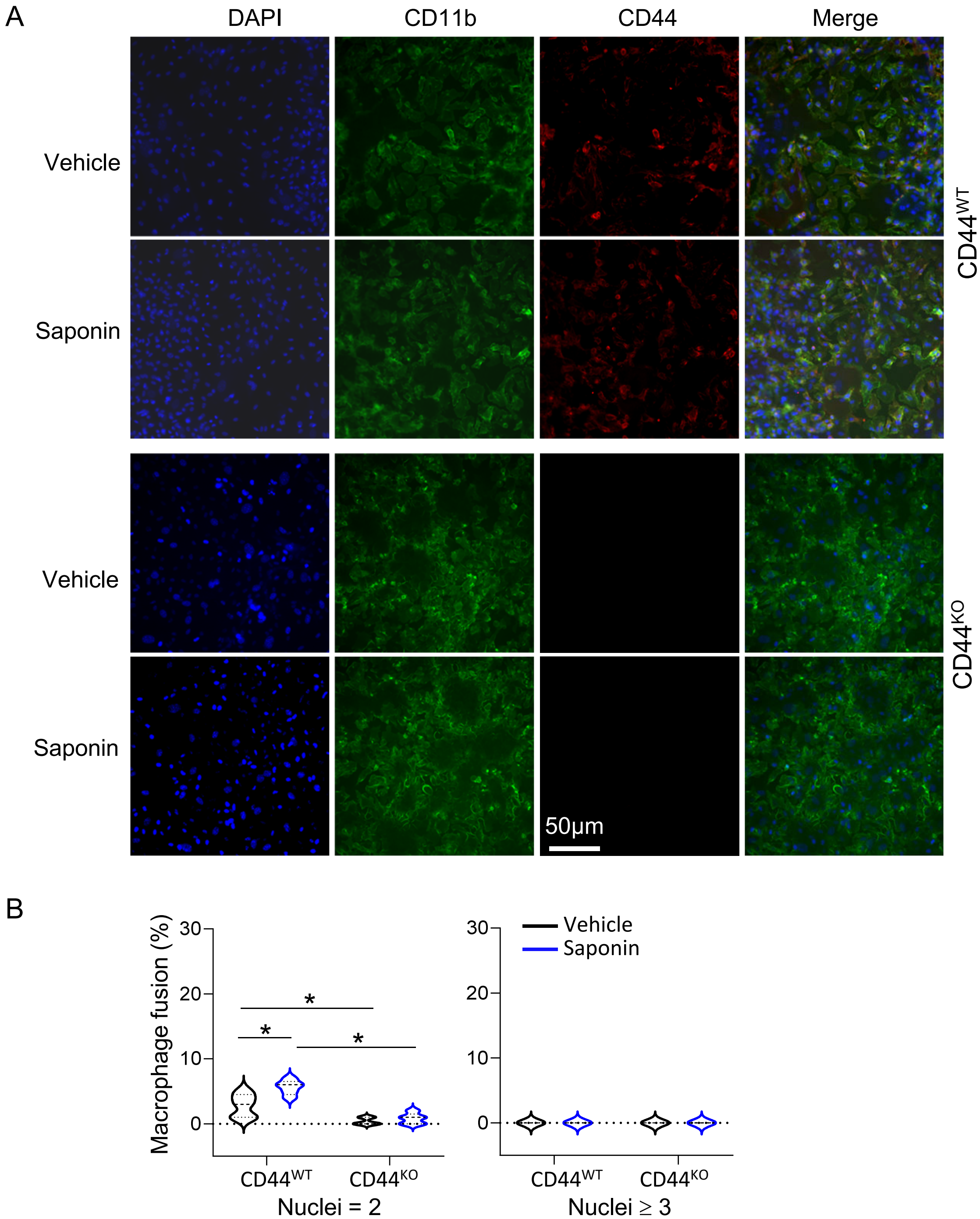

Supplemental Figure 7. Effects of saponin on macrophage fusion in the absence of IL-4. (A) Representative fluorescent images of *Cd44*-WT (Upper) or *Cd44*-deficient (Lower) BMDMs treated with saponin or the vehicle in the absence of IL-4. Images were acquired using Cellomics. (B) Statistical analysis of macrophage fusion in (A). One hundred cells (n = 100) were assessed in each sample. Percentages of cells with 2 or ≥3 nuclei were depicted. ANOVA test, *$p < 0.05$. Data shown are representative of 2 independent experiments.

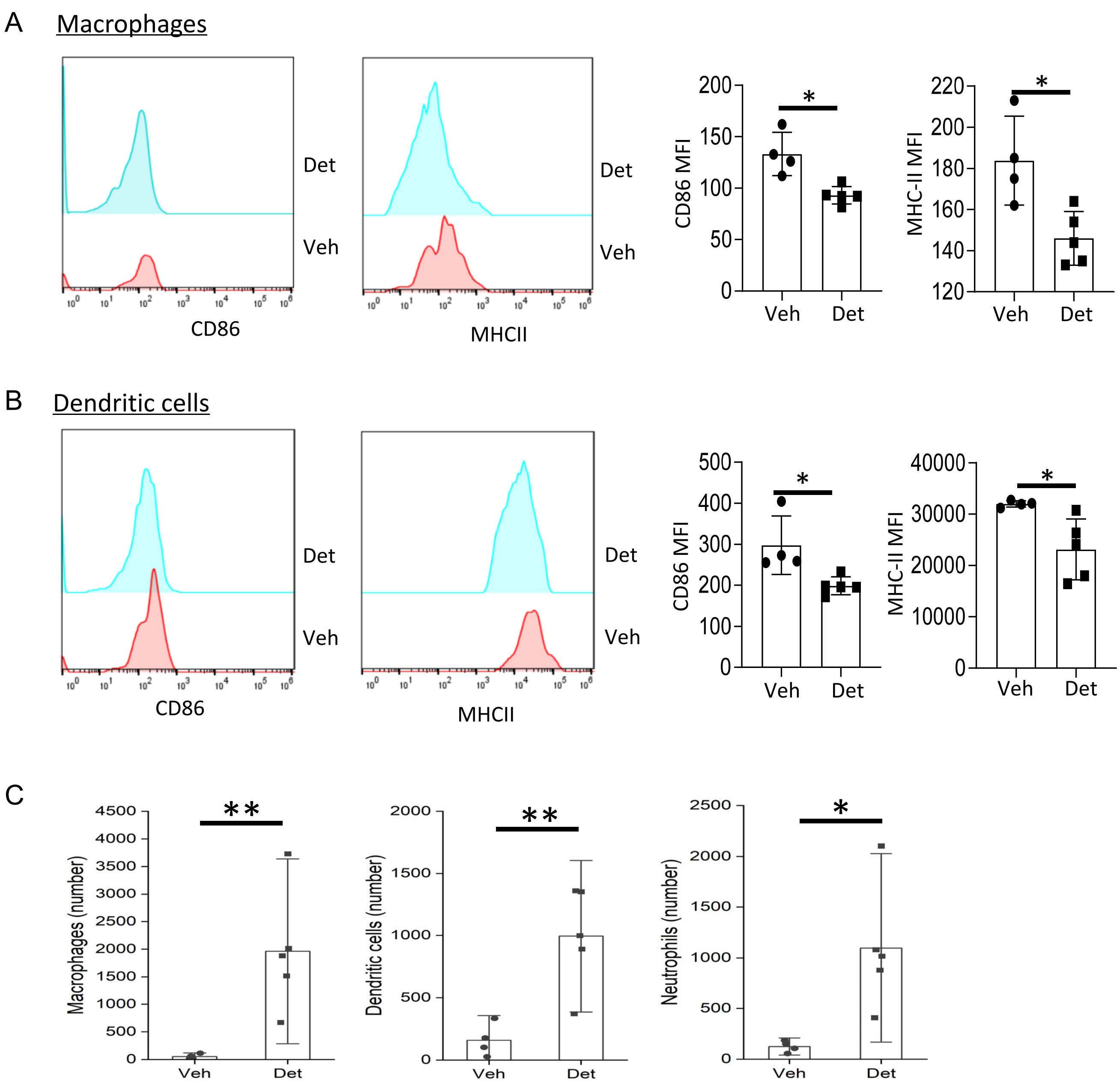

Supplemental Figure 8. Laundry detergent impairs antigen presentation and costimulatory function of dendritic cells and macrophages in mice. C57BL/6 mice were intratracheally (i.t.) instilled with laundry detergent or PBS once daily for four consecutive days, and were analyzed the following day. (A) Flow cytometry of MHC-II and CD86 on macrophages on a $CD11b^{+}Cd11c^{lo}$ gate. (B) Flow cytometry of MHC-II and CD86 on dendritic cells on a $CD11b^{+}Cd11c^{hi}$ gate. (C) Profiles of myeloid cells in BALF. Veh, vehicle; Det, detergent. Data (mean ± SD) are representative of two experiments. n = 4-5 per group. Student's t-test, $*p < 0.05$, $**p < 0.005$.
